# Spinal fusion achieves similar two-year improvement in HRQoL as total hip and total knee replacement. A prospective, multicentric and observational study

**DOI:** 10.1051/sicotj/2019027

**Published:** 2019-07-30

**Authors:** Javier Cervera Irimia, Félix Tomé-Bermejo, Angel R. Piñera-Parrilla, Marina Benito Gallo, Michele Bisaccia, Manuel Fernández-González, Julio Villar-Pérez, Jose Manuel Fernández-Carreira, Javier Orovio de Elizaga, Francisco Javier Areta-Jiménez, Luis Álvarez Galovich, Giuseppe Rollo, Luigi Caruso, Luigi Meccariello

**Affiliations:** 1 Department of Spine, Fundación Jiménez Díaz University Hospital Av. de los Reyes Católicos, 2 28040 Madrid Spain; 2 Department of Orthopaedic Surgery and Traumatology, Villalba General Hospital Carretera de Alpedrete a Moralzarzal M-608 Km 41 28400 Madrid Spain; 3 Division of Orthopedics and Trauma Surgery, University of Perugia, “S. Maria della Misericordia Hospital”, S. Andrea delle Fratte Piazzale Giorgio Menghini, 1 06129 Perugia Italy; 4 León University Health Care Centre Campus de Vegazana, s/n 24071 León Spain; 5 Research Support Unit, San Agustín Hospital Camino de Heros, 6 33401 Avilés Spain; 6 Catalonian General Hospital Carrer Pedro i Pons, 1, Sant Cugat del Vallés 08195 Barcelona Spain; 7 Gómez Ulla Central Armed Forces Hospital Glorieta Ejército, 1 28047 Madrid Spain; 8 Department of Orthopedics and Traumatology, Vito Fazzi Hospital Block A – V Floor, Piazzetta Muratore 73100 Lecce Italy

**Keywords:** Hip arthroplasty, Knee arthroplasty, Spinal surgery, Quality of life, Functional assessment

## Abstract

*Introduction*: Total hip and knee arthroplasty (THA/TKA) are surgical procedures with proven benefits. Although the literature reports outcomes of **fusion of the lumbar spine** comparable to those of THA/TKA in general health-related quality-of-life (HRQoL) questionnaires, functional assessment is nevertheless needed for these results to be of use in clinical practice and management. **Aim of our study was to prove** that lumbar spinal fusion has similar if not better outcomes than THA/TKA using intervention-specific HRQoL questionnaires and functional assessment questionnaires.

*Materials and methods*: Observational, ambispective, multicentre study of three cohorts undergoing lumbar spinal fusion (*n* = 115), THA (*n* = 119) and TKA (*n* = 253). Patients were evaluated using the Short-Form-12 (SF-12), Harris–Hip-Score, Hospital for Special Surgery Scale (HSS) and Oswestry Low Back Pain Disability questionnaires. A minimum follow-up of two years was conducted.

*Results*: The SF-12 showed significant improvement in all groups. The SF-12 physical component summary score indicated a more severe pre-operative status (*p* = *0.031*) in the THA cohort. The mental component summary score indicated a less severe pre-operative status in the TKA cohort (*p* = *0.008*) and greater post-operative improvement in the TKA and THA cohorts across follow-up (six months *p* = *0.021*; one year *p* = *0.012*; two years *p* = *0.042*). Functional assessment indicated greater pre-operative disability in the THA group. At two years of follow-up, functional improvement according to the Harris, HSS and Oswestry questionnaires were 152.01%, 50.07% and 41.14% respectively.

*Conclusions*: This study demonstrates that lumbar spinal fusion and total knee and hip arthroplasty are comparable in terms of functional improvement when thoroughly studied with health, quality-of-life and functional assessment questionnaires.

## Introduction

Due to population ageing and increased life expectancy, there has been a progressive increase in the prevalence of degenerative diseases [1, 2]. In developed countries some of the principal public health problems among older adults derive from lumbar, hip and knee pain, and are closely related to the presence and progression of arthritis [3, 4]. In the event of failure of conservative treatment, currently established surgical treatment for arthritis of hip and knee implies the use of replacement arthroplasty, while the management of degenerative disc disease entails the use of fusion techniques [5].

Arthroplasty of load-bearing joints, such as the hip and knee, is a widespread surgical treatment, and most authors agree on the fact that its functional benefits considerably outweigh both the risks and cost of the intervention [6–8]. Lumbar spinal fusion (arthrodesis) is a procedure performed to relieve pain by eliminating a pathological mobility [5]. Its indications, efficacy and cost-effectiveness appear to be in doubt, however, and many authors question its benefits as against those of standard conservative treatments [5, 9–13].

The real impact of lumbar spinal fusion on degenerative disease of the spine continues to be a source of controversy [14, 15]. Many aspects of this treatment are challenged and, in terms of evidence and cost-effectiveness, it is regarded as being inferior to selective hip or knee arthroplasty [12–14]. As a result, joint replacement surgery currently enjoys wide acceptance as a benchmark for assessing patient recovery in terms of an improvement in quality of life [16, 17]. Lumbar spinal fusion on the other hand, despite the substantial increase in spinal surgery rates in recent years, has not achieved the same degree of acceptance [18].

Many studies report unquestionably good results in terms of assessment of fusion rates and radiographic evidence of spinal surgery. Even so, some of these reviews report that patients’ opinions about their clinical results might not necessarily agree with those of their surgeons [19, 20]. It is currently acknowledged that, when it comes to assessing the outcome of any given surgical technique, one should examine, not merely the radiological and clinical outcome, but also patients’ expectations and variations in the perception of their health and functional status, since the two do not always coincide [21, 22].

There are a few studies in the literature, which compare the outcome of surgical treatment of spinal stenosis to that of hip and knee arthroplasty **[**23–25**].** While these studies report comparable results when using general health-related quality-of-life (HRQoL) questionnaires, they nevertheless lack the necessary intervention-specific functional disability assessment measures that would render the results useful for clinical practice and management. The use of functional assessment instruments makes it possible to detect the specific needs of each disease that are not taken into account in general health measurements [23–25].

Accordingly, the aim of our study was to assess the outcomes of patients who underwent instrumented lumbar spinal fusion for degenerative spondyloarthropathy and compare them to total hip and knee arthroplasty (THA/TKA), in terms of function and quality of life in the Spanish population. The study reports sequential results pre- and post-treatment in each cohort intervened, by assessing the changes and improvements in each cohort, with validated tools designed to measure general health status and with intervention-specific tools designed to assess patients’ functional status, and then comparing the outcomes of the three procedures.

## Patients and methodology

### Study design

An observational, ambispective, multicentre, cohort follow-up study was conducted in collaboration with hospitals in the different regions of Spain, which represented a wide spectrum of the country’s cultural variability. The study data were prospectively obtained in each group of patients and analysed retrospectively.

The study population consisted of patients drawn from twenty representative hospitals belonging to the National Health System (*Sistema Nacional de Salud*) and having different health-care levels and volumes of clinical activity. The respective Ethics Committees of the participating institutions approved the study protocol. The sample design was based on a convenience selection of health centres. Patients were selected at the various health centres in accordance with their respective volumes of clinical activity. We chose consecutive patients diagnosed with degenerative diseases of the spine, hip and knee. All patients were informed of the study’s objectives and the confidentiality of any data supplied, and they gave their consent to participate. All the patients included in the study underwent surgical interventions. Selected patients were assessed prior to the surgical intervention and underwent a protocoled follow-up at six months, one year and two years, with repetition of all questionnaires.

### Study subjects and data assessment

Three study groups, denominated “Hip”, “Knee” and “Lumbar”, were established for inclusion of patients who appeared on the scheduled surgery waiting list for total hip arthroplasty, total knee arthroplasty and instrumented lumbar spinal fusion respectively.

In the Hip and Knee groups, we included all patients who had long-term symptomatic hip or knee arthritis respectively, and had not responded to conservative treatment. Patients presenting with signs or symptoms that led to a suspected origin of pain other than primary arthritis were not included. The following were excluded from the study: patients aged under 55 years; patients who had undergone previous surgical interventions on the relevant joint (except arthroscopy) or revision surgery of a primary arthroplasty and patients with a rheumatic disease or any other inflammatory cause of the symptoms.

Those included in the Lumbar group were patients with diagnosis of spinal canal stenosis due to long-term degenerative spondyloarthropathy (spinal stenosis, degenerative disc disease or degenerative spondylolisthesis), who had not improved with conservative treatment and were scheduled for one or two levels of arthrodesis. Instrumented spinal fusion was indicated by a combination of variety of clinical factors (age, physiological status, or medical comorbidities), and anatomical findings. In the presence of preoperative instability (4 mm of translation or >10° of angular motion between adjacent endplates on lateral flexion and extension radiographs or spondylolistesis), or when Iaminectomy was accompanied by greater than 50% resection of both facets or complete facetectomy of one side, instrumented fusion was indicated. Patients presenting with signs or symptoms that led to a suspected origin of pain other than mechanical and degenerative were not included. The following were also excluded from the study: patients under the age of 55 years; and patients with previous lumbar surgery, rheumatic disease or some other specific diagnosis prior to the cause of their low back pain (i.e., scoliosis, congenital spinal disorders, neoplastic disease, infection or trauma). Patients with initially coexistent or developed during follow-up hip/knee/lumbar pathology were not included in the study.

### Assessment tools

We collected data on patients’ affiliation, sex, age and anthropometric features using a protocoled questionnaire; we measured health status using the Short Form-12 Physical Functioning (SF-12) questionnaire and its specific physical- and mental-component summary scores; and we measured impact on quality of life using specific instruments, such as the Harris Hip Score (HHS) and Hospital for Special Surgery Scale (HSS) questionnaires for assessing functional outcomes after implementation of hip and knee arthroplasties respectively, and the Oswestry Disability Index (ODI) for assessing disability associated with low back pain.

The SF-12 questionnaire is an HRQoL measurement tool made up of a set of 12 questions. It is a shortened, validated version of the SF-36, and is one of the most widely used general instruments in epidemiological and clinical research, whose psychometric properties have been evaluated in many studies. In addition, the SF-12 affords the advantage of furnishing specific physical-component (PCS-12) and mental-component summary (MCS-12) scores. The scores can be standardised in a range of 0–100, such that the higher the score, the better the patient’s HRQoL [26].

The ODI is a functional assessment questionnaire designed to assess disability associated with lumbar problems, i.e., to analyse the effects of low back pain on patients’ functional status. It is the “gold standard” of low back pain scales. It contains ten sections referring to activities of daily living. The total score is expressed as a percentage (from 0 to 100%), such that the higher the value, the greater the functional limitation of the patient [27].

The HHS is the most widely used instrument for assessing functional outcomes after hip arthroplasty. It includes four domains, namely, pain, function, range of motion and absence of deformity. The score is based on information gathered from patients’ interview and physical-examination results [28], and ranges from 0 (worst possible functional capacity) to 100 (best possible functional capacity).

The HSS is a functional assessment tool for assessing knee arthroplasty. The score is divided into seven categories, namely, pain, function, range of motion, muscle strength, flexion deformity, instability and subtractions. The HSS score takes one hundred points as its assessment base, such that the higher the score the better the outcome [29].

### Statistical analysis

Socio-demographic and control variables were described using analysis of frequency and proportions for the qualitative variables, and numerical summaries with measurements of central trend and dispersion for the quantitative variables. In the latter case, the Kolmogorov–Smirnov test was performed to ascertain the fit of the quantitative variables to a normal distribution. The populations of the three study groups were compared, using the Chi-squared or analysis of variance (ANOVA) test where applicable.

Overall mean pre- and post-surgery SF-12 and functional scale scores were compared in a protocoled manner among the different groups using ANOVA (with data drawn from the Oswestry questionnaire being converted into proportional inverse values for comparison with the other two functional scales). Similarly, PCS-12 and MCS-12 scores were independently compared. We also calculated the percentage improvement (analysed value – pre-operative value/post-operative value) in SF-12 and functional scale scores before and after surgery.

For study purposes, a *p*-value of 0.05 or under was deemed to be significant. All statistical analyses were performed using the IBM SPSS Statistics for Windows (2013), Version 22.0. (Armonk, NY: IBM Corp.)

## Results

In August 2010 the number of patients who were diagnosed of degenerative diseases of the spine, hip or knee and appeared on the scheduled surgery waiting list of the involved hospitals was 675. Thirty-nine patients declined to enter the study. One hundred and ten patients did not complete the study questionnaires during follow-up due to patient non-compliance with medical examinations or lack of information required for proper completion of health questionnaires. Thirty-seven patients were lost to follow-up, and two patients died from an underlying medical condition unrelated to the study. Ultimately, data from 487 patients (72.14%) were analysed.

The study analysed data drawn from 119 total hip arthroplasties (Hip group), 253 knee arthroplasties (Knee group), and 115 lumbar spinal fusions (Lumbar group). The Hip group included 92 (77.31%) patients with diagnosis of **osteoarthritis**, 25 (21.01%) with avascular necrosis, and two with hip dysplasia (1.68%). In the Knee group patients with diagnosis of arthritis were the only ones included. And lastly, in the lumbar spinal fusion group there were included 39 (33.91%) patients with degenerative disc disease, 60 patients (52.17%) with spinal stenosis, and 16 patients (13.91%) with spinal instability.

**Figure 1:**
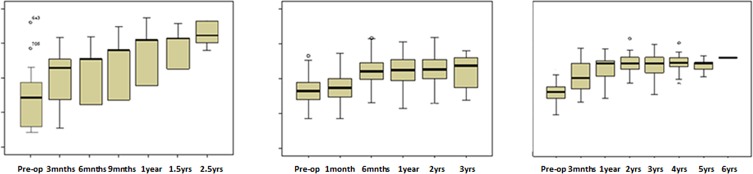
Trends in the SF-12 physical-component summary (PSC) score during follow-up of the Lumbar, Knee and Hip cohorts.

The demographic and anthropometric data of the three cohorts are shown in Table 1. Table 2 provides an analysis of the general perception of patients’ health status across follow-up, by reference to a detailed breakdown of the three cohorts’ PCS-12 and MCS-12 scores on the SF-12 health questionnaire. Comparison of cohorts’ PCS-12 scores for pre-operative data indicated a more severely limited function pre-operatively for Hip group (30.20%) than for Knee (33.65%) and Lumbar group (33.53%) patients, with this difference among the patients of the three cohorts proving statistically significant (*p* = 0.032). During follow-up, PCS-12 scores also revealed that the Hip group patients displayed a higher final percentage improvement (43.52–30.20 = 13.32%) than did the remaining cohorts in the Knee (44.32–33.65 = 10.67%) and Lumbar groups (44.61–33.53 = 11.08%). When this difference was analysed, however, it proved to be statistically non-significant (PCS Hip group improvement vs. Knee and Lumbar groups’ improvement: six months *p* = 0.845; one year *p* = 0.644; two years: *p* = 0.933) (Figure 1).

**Table 1:** Population characteristics – age, body mass index (BMI) and sex.

Group	Age	BMI	Sex	
Lumbar	67.08 ± 6.71	28.22 ± 4.32	♀61	♂54
Hip	66.26 ± 7.30	27.79 ± 3.72	♀72	♂47
Knee	71.95 ± 6.28	30.30 ± 4.32	♀190	♂63

**Table 2:** Outcome variables – SF12 – PCS12 and MCS12.

	Group	Preoperative	Six months	One year	Two years
PCS12	Lumbar	33.5248	42.6614	41.7181	44.6080
	Hip	30.2020	44.5914	43.8825	43.5217
	Knee	33.6488	43.9862	44.3401	44.3233
		*p* = *0.032*	*p* = *0.845*	*p* = *0.644*	*p* = *0.933*
MCS12	Lumbar	40.1949	49.3054	51.3473	53.0640
	Hip	41.9455	53.5451	54.1536	58.4307
	Knee	46.3444	48.3779	50.4471	50.4793
		*p* = *0.008*	*p* = *0.022*	*p* = *0.012*	*p* = *0.043*

In terms of MCS scores, Knee group patients were observed to have a statistically significant less severe pre-operative status (*p* = *0.008*) and, though a significant improvement was observed for all cohorts across follow-up, the improvement was nonetheless greater among the Lumbar and Hip group patients than among those in the Knee group. Moreover, this difference proved to be statistically significant for all follow-up periods (Hip and Lumbar groups’ improvement **with respect** to Knee group improvement: six months *p* = 0.022; one year *p* = 0.012; two years *p* = 0.043).

Analysis of patients’ functional questionnaires on the different assessment scales (Harris, HSS and Oswestry) showed a clear functional improvement in all three groups post-surgery and across follow-up (Table 3). Patients who underwent hip replacement surgery registered a percentage functional improvement (Table 4) of 152.01% (from 36.11 to 91.00) at two years according to the Harris scale; those who underwent knee replacement surgery registered a functional improvement of 50.07% (from 56.42 to 84.67) according to the HSS scale; and those who underwent spinal surgery registered a functional improvement of 41.14% (from 58.73 to 82.89) at two years according to the Oswestry scale. As in the case of the SF-12 health questionnaire, Hip group patients presented with greater disability prior to the surgical intervention, as well as greater improvement in functional status across follow-up, with this superiority in functional improvement proving statistically significant. The functional improvement observed for patients in the Knee and Lumbar groups was very similar (six months *p* = 0.027; one year *p* = 0.278; two years: *p* = 0.302).

**Table 3:** Outcome variables – functional scales.

	Preoperative			Six months			12 months			24 months		
Scales	OSW	Harris	HSS	OSW	Harris	HSS	OSW	Harris	HSS	OSW	Harris	HSS
Average	58.73	36.11	56.42	83.00	88.81	81.21	81.95	88.26	83.88	82.89	91.00	84.67
Standard deviation	22,046	12,374	12,008	18,163	5680	11,997	14,564	7241	10,882	11,742	3789	8867
ANOVA	*P* < 0.001			*P* = 0.027			*P* = 0.278			*P* = 0.302		

OSW: Oswestry (Lumbar); Harris (Hip); HSS (Knee).

ANOVA, analysis of variance; HSS, Hospital for Special Surgery Scale.

**Table 4:** Outcome variables – functional-scale improvement percentages.

Functional improvement (%)	Six months	One year	Two years
Oswestry	41.33%	39.54	41.14%
Harris	145.94%	144.42%	152.01%
HSS	43.94%	48.67%	50.07%

ANOVA, analysis of variance; HSS, Hospital for Special Surgery Scale.

## Discussion

This study shows that lumbar spinal fusion is capable of producing substantial and significant improvements in the health and functional status of patients undergoing surgery and in terms of results, it is also a comparable procedure to arthroplasties of major load-bearing joints, and that it achieves clinical outcomes similar to those of hip and knee arthroplasties, which are the archetypical benchmarks of successful surgical treatment.

Traditionally, the scientific literature has highlighted the existence of a significant variability in clinical outcomes obtained by patients undergoing lumbar spinal fusion **[14]**. This fact contrasts with existing data of low-intermediate variability in hip and knee replacement surgery [30]. Similarly, the literature also reflects a lack of consensus on the indications for justifying lumbar spinal fusion in terms of results. These data have fostered the idea of a lack of scientific evidence of the efficacy and cost-effectiveness of lumbar spinal fusion [9, 14, 18, 31].

Depending on the diagnostic criterion used, and the age and sex of the population considered, there is great geographical variability in the estimated prevalence of arthritis. In Spain, data supplied by the Spanish Society of Rheumatology (*Sociedad Española de Reumatología*) show that there are over seven million people with knee, hand or spine degenerative diseases, which annually cost the health authorities around 4800 million euros, 0.5% of Spain’s **Gross Domestic Product (GDP)** [32]. According to the World Health Organisation, **about** 28% of the world’s population over the age of 60 years suffer from osteoarthritis, and 80% of them have restricted mobility. The increasing life expectancy and ageing of population means that arthritis may become the fourth leading cause of disability by 2020 [30]. The growing body of evidence indicating that spinal surgery is an effective treatment for patients with failure of conservative treatment has translated into an increased demand for and acceptance of spinal surgery. The results of this study have direct implications on health care and policy from a patient standpoint.

This nation-wide multicentre study shows an objective appraisal of the impact of surgical procedures performed to improve the wellbeing of patients affected by degenerative diseases of the spine, hip and knee. Our primary aim was to assess the comparable outcomes of lumbar spine fusion versus hip and knee surgery treatment both comprehensively and from the patients’ perception, on the basis of a set of clinical and functional self-assessment tools validated for measuring health status and functional status by reference to standard domains of assessment and physical examination [23–25]. Thus, it’s been possible to compare three different degenerative-disease surgeries by using identical, specific methods to analyse their results.

One aspect that is not taken into account when health questionnaires are used as the sole method of assessment is analysis of patients’ functional status. Standards for measurement of outcomes in clinical research for orthopaedic disorders include recommendations for the SF-12 or the EQ-5D to measure general health [33]. Serious trade-offs involved in choosing outcome measures for clinical trials include the need to obtain adequate power to detect differences and to maximise measurement precision. With these concerns in mind, investigators often favour disease-specific measurements that appear to focus on the key aspects targeted by treatment over generic preference-based instruments [34]. This study incorporated functional disability assessment scales that were specific to each intervention, to ensure that the results would be useful in clinical practice and management. The use of functional assessment instruments allows us to detect those needs that might otherwise not be borne in mind if health measurements alone were used [21, 22]. The scale scores for these groups of patients showed that those who underwent hip arthroplasty presented with greater disability before surgical intervention and better functional status across follow-up than did the other two cohorts studied.

This study used an appropriately selected sample of the Mediterranean population, taking the area’s specific socio-economic/occupational variability into account. It shows that the results obtained in lumbar spinal fusion surgery are as effective as those achieved in knee and hip arthroplasty surgery, and that these improvements are maintained for a minimum of two years after the intervention. This finding confirms the conclusions reached by other earlier studies. In surgical treatment of spinal stenosis, Rampersaud et al. [35] examined HRQoL at two years using the SF-36 questionnaire. Their study furnished results similar to ours, but its analysis incorporated a mix of spinal surgery procedures with and without fusion. In another subsequent study [36], the same authors reported equally comparable results for the three procedures even after a seven-year follow-up, despite the finding of a higher re-intervention rate in patients who underwent spinal surgery than in the other two cohorts.

In a similar study, Juul et al. [37] reported comparable improvements in SF-36 results at one year between patients who underwent hip arthroplasty and others treated with lumbar spinal fusion. Added to that, the group of patients who underwent hip arthroplasty displayed a higher improvement percentage than did the remaining cohorts that were intervened. Both percentage domains – health and functional status – increased more in patients who underwent hip arthroplasty than in the other cohorts, and it was the same for the correlation between the Harris scale and the SF-12 questionnaire. These results might be related to the fact that these patients started from a worse baseline situation, and would thus have a tendency to show greater improvement in their outcomes. Another study conducted by Hansson et al. [38] reported similar results than ours, with a greater improvement among patients who underwent hip surgery, although different spinal surgical techniques (with and without instrumentation and fusion) were included.

### Study limitations

Some of the previous authors report that patients with a more severe pre-operative physical status would experience a smaller post-operative improvement [39]. After analysing the results of our series, we observed that this relationship could in fact be more complex. A more deteriorated pre-operative condition could influence the quality of life expected after surgery. Similarly, patients in our study diagnosed with knee osteoarthritis presented with a less severe pre-operative status, and yet also registered poorer results during follow-up, particularly in mental-scale scores.

It should also been mentioned those limitations linked to the design of the study itself as an observational, ambispective, multicentre and cohort follow-up one. Primarily, we overlooked the role of some factors, such as surgical anaesthetic risk or pre-existing comorbidities, which intervene in the results when it comes to analysing improvement in quality of life, and could thus be modifying factors. Secondly, with regard to the sample and its representativeness, it should likewise be noted that there were considerable losses to follow-up. Among the causes of such losses, the most important was the difficulty of recruiting and following-up patients with indication of lumbar spinal fusion, for study purposes. This limitation has been attributed to demographic changes in the catchment area, the influence of occupational disability allowances and/or difficulties in absorbing the considerable volume of spinal surgery currently on waiting lists [40]. This situation, along with the fact that some health centres recruited fewer cases than expected, may have given rise to selection bias.

Our use of the SF-12 questionnaire was due to the fact that it affords an invaluable method of comparison for measuring the effectiveness of treatments in different diseases; to its widespread use, which allows for comparison with other studies; and to its good discriminatory capacity for detecting variations in health status. However, the SF-12 has a lower power than the extended version SF-36, with the same shortcomings of neither evaluating nor assessing symptoms associated with the disease, and remaining insensitive to changes in specific conditions, as would be expected from a more specific instrument [26]. In order to avoid selection and follow-up biases, the following should be borne in mind in future studies: comparison to other groups, such as healthy patients, inclusion of control groups and stratification by specific diseases.

Finally, we must mention that the two-year follow-up period should have been ideally longer since it is possible to detect other differences in longer-term results that we might have missed.

The results of this study allow us to conclude, in terms of improvement in quality of life and function, that lumbar fusion surgery achieves similar results to those of hip and knee arthroplasty surgery. In patients diagnosed with degenerative spondyloarthropathy, lumbar spinal fusion achieves an improvement in general wellbeing and physical capacity, comparable to that yielded by total knee arthroplasty and slightly inferior to that achieved by total hip arthroplasty.

We recommend the use of the mentioned scales to assess health, quality of life and functional status in routine clinical practice and decision-making, as indispensable methods for assessment and follow-up with a view to drawing up future treatment guidelines.

## Human and animal right

Any statement relating to studies on humans and animals is not required for this type of study. All patients gave the informed consent prior to being included into the study. All procedures involving human participants were in accordance with the 1964 Helsinki declaration and its later amendments.

## References

[1] Deyo RA, Gray DT, Kreuter W, Mirza S, Martin BI (2005) United States trends in lumbar fusion surgery for degenerative conditions. Spine 30, 1441–1445. 15959375 10.1097/01.brs.0000166503.37969.8a

[2] Kurtz S, Mowat F, Ong K, Chan N, Lau E, Halpern M (2005) Prevalence of primary and revision total hip and knee arthroplasty in the United States from 1990 through 2002. J Bone Joint Surg Am 87, 1487–1497. 15995115 10.2106/JBJS.D.02441

[3] Dawson J, Linsell L, Zondervan K, Rose P, Carr A, Randall T, Fitzpatrick R (2005) Impact of persistent hip or knee pain on overall health status in elderly people: A longitudinal population study. Arthritis Rheum 53, 368–374. 15934104 10.1002/art.21180

[4] Muraki S, Oka H, Akune T, Mabuchi A, En-Yo Y, Yoshida M, Saika A, Suzuki T, Yoshida H, Ishibashi H, Yamamoto S, Nakamura K, Kawaguchi H, Yoshimura N (2009) Prevalence of radiographic lumbar spondylosis and its association with low back pain in the elderly of population-based cohorts: The ROAD study. Ann Rheum Dis 68, 1401–1406. 18718988 10.1136/ard.2007.087296

[5] Deyo RA, Nachemson A, Mirza SK (2004) Spinal fusion surgery the case for restraint. N Engl J Med 350(7), 722–726. 14960750 10.1056/NEJMsb031771

[6] Harris WH, Sledge CB (1990) Total hip and total knee replacement (1). N Engl J Med 323, 725–731. 2201916 10.1056/NEJM199009133231106

[7] Harris WH, Sledge CB (1990) Total hip and total knee replacement (2). N Engl J Med 323, 801–807. 2136367 10.1056/NEJM199009203231206

[8] Liang MH, Cullen KE, Poss R (1982) Primary total hip or knee replacement: Evaluation of patients. Ann Intern Med 97, 735–739. 6753682 10.7326/0003-4819-97-5-735

[9] Gibson JN, Grant IC, Waddell G (1999) The Cochrane review of surgery for lumbar disc prolapse and degenerative lumbar spondylosis. Spine 24, 1820–1832. 10488513 10.1097/00007632-199909010-00012

[10] Keller RB (1993) Outcomes research in orthopaedics. J Am Acad Orthop Surg 1, 122–129. 10675863 10.5435/00124635-199311000-00007

[11] Fairbank J, Frost H, Wilson-MacDonald J, Yu LM, Barker K, Collins R, Spine Stabilisation Trial Group (2005) Randomised controlled trial to compare surgical stabilisation of the lumbar spine with an intensive rehabilitation programme for patients with chronic low back pain: The MRC spine stabilisation trial. BMJ 330, 1233–1239. 15911537 10.1136/bmj.38441.620417.8F PMC558090

[12] Nachemson A, Zdeblic TA, O’Brian JP (1996) Lumbar disc disease with dicogenic pain. What surgical treatment is most effective? Spine 21(15), 1835–1838. 8855471 10.1097/00007632-199608010-00023

[13] Hägg O, Fritzell P, Nordwall A, The Swedish Lumbar Spine Study Group (2002) Characteristics of patients with low back pain selected for surgery: A comparison with the general population reported from the Swedish lumbar spine study. Spine 27(11), 1230–1231. 12045521 10.1097/00007632-200206010-00015

[14] Turner JA, Ersek M, Herron L (1992) Surgery for lumbar spinal stenosis. Attempted meta-analysis of the literature. Spine 17, 1–8. 1531550 10.1097/00007632-199201000-00001

[15] Katz JN (1998) Cost-effectiveness of spine surgery: The jury is out. Ann Intern Med 149, 901–903. 10.7326/0003-4819-149-12-200812160-00010 19075210

[16] Ethgen O, Bruyere O, Richy F, Dardennes C, Reginster JY (2004) Health-related quality of life in total hip and total knee arthroplasty. A qualitative and systematic review of the literature. J Bone Joint Surg Am 86, 963–974. 15118039 10.2106/00004623-200405000-00012

[17] Malchau H, Garellick G, Eisler T, Kärrholm J, Herberts P (2005) Presidential guest address: The Swedish Hip Registry: Increasing the sensitivity by patient outcome data. Clin Orthop Relat Res 441, 19–29. 16330981 10.1097/01.blo.0000193517.19556.e4

[18] Weinstein JN, Lurie JD, Olson PR, Bronner KK, Fisher ES (2006) United States’ trends and regional variations in lumbar spine surgery: 1992–2003. Spine 31, 2707–2714. 17077740 10.1097/01.brs.0000248132.15231.fe PMC2913862

[19] Saban Karen L, Penckofer Sue M, Androwich Ida, Bryant Fred B (2007) Health-related quality of life of patients following selected types of lumbar spinal surgery: A pilot study. Health Qual Life Outcomes 5, 71. 18163905 10.1186/1477-7525-5-71 PMC2246115

[20] Polly DW Jr, Glassman SD, Schwender JD, Shaffrey CI, Branch C, Burkus JK, Gornet MF, Lumbar Spine Study Group (2007) SF-36 PCS benefit cost ratio of lumbar fusion comparison to other surgical interventions: A thought experiment. Spine 32(11 Suppl), S20–S26. 17495582 10.1097/BRS.0b013e318053d4e5

[21] Rollfson O, Dahlberg LE, Nilsson JA, Malchau H, Garellick G (2009) Variables determining outcome in total hip replacement surgery. J Bone Joint Surg (Br) 91-B, 157–161. 10.1302/0301-620X.91B2.20765 19190046

[22] Vilagut G, Valderas JM, Ferrer M, Garin O, Lopez-Garcia E, Alonso J (2008) Interpretation of SF-36 and SF-12 questionnaires in Spain: Physical and mental components. Med Clin 130, 726–735. 10.1157/13121076 18570798

[23] Deyo RA, Battie M, Beurskens AJ, Bombardier C, Croft P, Koes B, Malmivaara A, Roland M, Von Korff M, Waddell G (1998) Outcome measures for low back pain research. A proposal for standardized use. Spine 23, 2003–2013. 9779535 10.1097/00007632-199809150-00018

[24] Bombardier C (2000) Outcome assessments in the evaluation of treatment of spinal disorders. Introduction. Spine 25, 3097–3099. 11124723 10.1097/00007632-200012150-00002

[25] Bombardier C (2000) Outcome assessments in the evaluation of treatment of spinal disorders: Summary and general recommendations. Spine 25, 3100–3103. 11124724 10.1097/00007632-200012150-00003

[26] Vilagut G, Valderas JM, Ferrer M, Garin O, Lopez-Garcia E, Alonso J (2008) Interpretation of SF-36 and SF-12 questionnaires in Spain: Physical and mental components. Med Clin 130, 726–735. 10.1157/13121076 18570798

[27] Fairbank JCT, Davies JB, Couper J, O’Brien JP (1980) The Oswestry low back pain disability questionnaire. Physiother 66, 271–273. 6450426

[28] Söderman P, Malchau H, Herberts P (2001) Outcome of total hip replacement: A comparison of different measurement methods. Clin Orthop Relat Res Sep(390), 163–172. 10.1097/00003086-200109000-00019 11550862

[29] Callahan CM, Drake BG, Heck DA, Dittus RS (1994) Patient outcomes following tri compartmental total knee replacement. A meta-analysis. JAMA 271, 1349–1357. 8158821

[30] Woolf AD, Pfleger B (2003) Burden of major musculoskeletal conditions. Bull World Health Organ 81(9), 646–656. 14710506 PMC2572542

[31] Hansson TH, Hansson EK (2000) The effects of common medical interventions on pain, back function and work resumption in patients with chronic low back pain: A prospective 2 year cohort study. Spine 25, 3055–3064. 11145817 10.1097/00007632-200012010-00013

[32] Carmona L, Villaverde V, Hernández-García C, Ballina J, Gabriel R, Laffon A, EPISER Study Group (2002) The prevalence of rheumatoid arthritis in the general population of Spain. Rheumatology (Oxford) 41(1), 88–95. 11792885 10.1093/rheumatology/41.1.88

[33] Chiarotto A, Terwee CB, Deyo RA, Boers M, Lin CW, Buchbinder R, Corbin TP, Costa LO, Foster NE, Grotle M, Koes BW, Kovacs FM, Maher CG, Pearson AM, Peul WC, Schoene ML, Turk DC, van Tulder MW, Ostelo RW (2014) A core outcome set for clinical trials on non-specific low back pain: Study protocol for the development of a core domain set. Trials 15, 511. 25540987 10.1186/1745-6215-15-511 PMC4308079

[34] Carreon LY, Glassman SD, McDonough CM, Rampersaud R, Berven S, Shainline M (2009) Predicting SF-6D utility scores from the Oswestry disability index and numeric rating scales for back and leg pain. Spine 34(19), 2085–2089. 19730215 10.1097/BRS.0b013e3181a93ea6 PMC3504506

[35] Rampersaud YR, Ravi B, Lewis SJ, Stas V, Barron R, Davey R, Mahomed N (2008) Assessment of health-related quality of life after surgical treatment of focal symptomatic spinal stenosis compared with osteoarthritis of the hip or knee. Spine J 8, 296–304. 17669690 10.1016/j.spinee.2007.05.003

[36] Rampersaud YR, Lewis SJ, Davey JR, Gandhi R, Mahomed N (2014) Comparative outcomes and cost-utility after surgical treatment of focal lumbar spinal stenosis compared with osteoarthritis of the hip or knee-part 1: Long-term change in health-related quality of life. Spine J 14, 234–243. 24325880 10.1016/j.spinee.2013.12.010

[37] Juul O, Sigmundsson FG, Ovesen O, Andersen MO, Ernst C, Thomsen K (2006) No difference in health-related quality of life in hip osteoarthritis compared to degenerative lumbar instability at pre- and 1-year postoperatively: A prospective study of 101 patients. Acta Orthop 77(5), 748–754. 17068705 10.1080/17453670610012935

[38] Hansson T, Hansson E, Malchau H (2008) Utility of spine surgery: A comparison of common elective orthopaedic surgical procedures. Spine 33, 2819–2830. 19050588 10.1097/BRS.0b013e31818e2914

[39] Rolfson O, Dahlberg LE, Nilsson JA, Malchau H, Garellick G (2009) Variables determining outcome in total hip replacement surgery. J Bone Joint Surg (Br) 91-B, 157–161. 10.1302/0301-620X.91B2.20765 19190046

[40] Birkmeyer JD, Sharp SM, Finlayson SR, Fisher ES, Wennberg JE (1998) Variation profiles of common surgical procedures. Surgery 124, 917–923. 9823407

